# Comparison of DNA and RNA sequencing of total nucleic acids from human cervix for metagenomics

**DOI:** 10.1038/s41598-021-98452-4

**Published:** 2021-09-22

**Authors:** Laila Sara Arroyo Mühr, Joakim Dillner, Agustin Enrique Ure, Karin Sundström, Emilie Hultin

**Affiliations:** 1 Department of Laboratory Medicine, Karolinska Institutet, 141 86 Stockholm, Sweden; 2 Clinical Pathology/Cytology, Karolinska University Laboratory, Karolinska University Hospital, 141 86 Stockholm, Sweden

**Keywords:** DNA sequencing, Next-generation sequencing, RNA sequencing, Tumour virus infections, Cervical cancer, Human papilloma virus, Metagenomics, Metagenomics, Microbiome, Metagenomics

## Abstract

Although metagenomics and metatranscriptomics are commonly used to identify bacteria and viruses in human samples, few studies directly compare these strategies. We wished to compare DNA and RNA sequencing of bacterial and viral metagenomes and metatranscriptomes in the human cervix. Total nucleic acids from six human cervical samples were subjected to DNA and RNA sequencing. The effect of DNase-treatment before reverse transcription to cDNA were also analyzed. Similarities and differences in the metagenomic findings with the three different sequencing approaches were evaluated. A higher proportion of human sequences were detected by DNA sequencing (93%) compared to RNA sequencing without (76%) and with prior DNase-treatment (11%). On the contrary, bacterial sequences increased 17 and 91 times. However, the number of detected bacterial genera were less by RNA sequencing, suggesting that only a few contribute to most of the bacterial transcripts. The viral sequences were less by RNA sequencing, still twice as many virus genera were detected, including some RNA viruses that were missed by DNA sequencing. Metatranscriptomics of total cDNA provided improved detection of mainly transcribed bacteria and viruses in cervical swabs as well as detection of RNA viruses, compared to metagenomics.

## Introduction

The number of bacterial cells within the human body is approximately the same as the number of human cells, 10^13^ in total^1^, and there are approximately 150 times more genes in the human microbiome compared to the human genome^2,3^. Viruses are also very abundant in the human body, at a similar level as the bacteria^4^, and they make up the virome. The human virome includes viruses that infect archaea and human cells, bacteriophages that infect bacteria, but also transient viruses. Amplicon sequencing is applicable to analyze organisms that share common genes, like prokaryotes, fungi and eukaryotes that all share common parts in their genomes. Bacterial species present in different specimens for example, are commonly identified using amplicon sequencing of the 16S ribosomal RNA gene^5^. However, viruses do not share any specific gene or other DNA/RNA sequence and hence, other sequencing approaches than amplicon sequencing are necessary to identify and study them.

Different viral target enrichment methods like filtration can be used prior massively parallel DNA sequencing in order to study the virome^6^. However, several disadvantages might exist when trying to enrich viruses in a biospecimen: (a) unknown viruses not targeted with the enrichment approach might escape identification, (b) a proportion of viruses might be lost depending on the enrichment method, (c) if the human genome is depleted in the sample in order to enrich for viruses, information regarding the human genome will not be included and possible viral integration may also fall undetected and, (d) RNA viruses will not be identified when using a DNA extraction/DNA sequencing approach.

Nowadays, one approach to study the whole virome (as well as the human genome) is based on sequencing all the DNA present in a specimen, so called shotgun DNA sequencing^7–9^. With this, all DNA viruses and other microbes present within a specimen (known and unknown) should be detected if sequencing is performed at sufficient depth. Similarly to shotgun DNA sequencing, massively parallel cDNA sequencing from total RNA can be used to explore the metatranscriptomes within a specimen in order to perform microbiome and virome profiling^10^ in addition to analysis for differential gene expression of both human, microbial and viral genes. Microbiome and virome profiling using RNA sequencing gives the additional information about actively transcribed genes and has also the possibility to detect viruses with RNA genomes, which cannot be detected by shotgun DNA sequencing if RNA is not reverse-transcribed first.

Cervical cancer is caused by persistent infection of oncogenic high risk human papillomavirus (HPV) types^11–13^. The most oncogenic HPV types that cause neoplasia and cancer of the cervix are HPV types 16 and 18, but other HPV types are also found in cervical neoplasia^14–16^. Cervical squamous intraepithelial neoplasia stage 3 (CIN3), cervical cancer in situ, may continue to develop into invasive cancer if not treated, but it is not entirely clear which factors are involved in the persistence of the infection and the progression into cancer or clearance of the infection. The vaginal microbiota has been characterized in both normal and cervical intraepithelial neoplasia as well as cervical cancer^17^. Metagenomic studies suggest that only one or a few species of *Lactobacillus* within the phylum Firmicutes are usually dominating the vaginal microbiota^18^. However, the diversity tends to increase with neoplasia and HPV-positivity^19^. The vaginal microbiota may have an important role to protect against harmful infections like for instance HPVs, hence, the cervical microbiota may serve as a potential biomarker for cancer progression risk^17,20,21^.

To eliminate cervical cancer, a combination of broad vaccination programs^22^ and early biomarkers with high specificity and sensitivity in order to identify women with low grade neoplasia with high risk of progression into cancer is important. In the present study, the aim was to compare massively parallel DNA and RNA sequencing of total nucleic acid extracted material for a comprehensive detection of the detectable and actively transcribing DNA and RNA microbes in cervical specimens.

## Material and methods

### Study participants

The sample collection of the Swedish Center for Cervical Cancer Prevention currently holds cervical samples from > 400,000 women, stored under validated methods in − 25 °C^23^. All women 23–64 years of age in this sample collection were diagnosed with CIN2 + (including CIN2, CIN3 = squamous cell carcinoma in situ, adenocarcinoma in situ and invasive cervical cancer) during the years 2013–2015 and identified by linkage to the National Cervical Screening Registry (NKCx), which also has the Center for Cervical Cancer Prevention as responsible organization. A random of 6 cervical samples (3 CIN3 + samples and 3 normal with no cervical disease diagnosed) were included for the present study from different women (n = 6). The CIN3 samples were matched with the normal samples by age in 5-year bands and calendar year of liquid-based cytology (LBC) test. Ethical approval was granted by the Regional Ethical Review Board of Stockholm, Sweden (EPN-Dnr: 2014/1242-31/4). Written informed consent was obtained from all participants. All methods were carried out in accordance with relevant guidelines and regulations.

### Sample preparation

Isolation of total nucleic acid from the 6 LBC swab samples was performed using MagNA Pure LC instrument and total nucleic acid isolation kit (Roche, Basel, Switzerland) in accordance with the manufacturer´s protocol (Roche Molecular Systems, Inc., Alameda, CA, USA). 100 ul were used for total nucleic extraction and total nucleic acid was eluted in 50 µL elution buffer. The extracted DNA concentration was quantified in all 6 total nucleic acid extracted samples using a fluorometric assay (QuantiFluorST, Promega, US) according to manufacturer´s instructions, and ranged from 0.08 to 2.9 ng/ul (average 1.04 ng/ul).

Total nucleic acid extracted material from the 6 samples was thereafter subjected to 3 different approaches to analyze the microbiota: metagenomic DNA sequencing, metatranscriptomic RNA sequencing and metatranscriptomic RNA sequencing including a step of DNase-treatment of the total nucleic acids before RNA reverse transcription to cDNA. In total, 18 DNA/cDNA libraries were sequenced.

### DNA approach: library preparation and sequencing

For DNA sequencing, DNA libraries were prepared directly from the total nucleic acid extracted material from the 6 cervical samples using Nextera XT DNA library preparation kit (Illumina, San Diego, CA, USA) following the manufacturers’ reference guide, starting with 1 ng of DNA (as recommended by the manufacturer) and using unique indexed adapters to facilitate pooling of the libraries. The 6 DNA libraries were normalized to 1 nM and pooled prior paired-end sequencing at 1.8 pM for 2 × 150 bp using NextSeq500 (Illumina, San Diego, CA, USA).

### RNA approach: library preparation and sequencing

For RNA sequencing, RNA libraries were prepared directly from the total nucleic acid extracted material (not RNA extracted material) from the 6 cervical samples using Smarter stranded total RNA-seq kit v2—pico input mammalian (Takara Bio USA, Mountain View, CA, USA) following the user manual. Starting material was 8 ul as recommended in the guidelines, prior fragmentation for 2 min. After cDNA synthesis, adapters including an index part, unique for each individual sample, were added to the cDNA fragments, following ribosomal cDNA depletion and enrichment of uncleaved fragments by 14 cycles of PCR. The 6 RNA libraries were then normalized to 4 nM and pooled prior paired-end sequencing at 1.2 pM for 2 × 75 bp using NextSeq500 (Illumina, San Diego, CA, USA).

### RNA with DNase-treatment approach: library preparation and sequencing

To observe if there was an effect of DNA presence in RNA sequencing, another 6 RNA libraries were prepared and sequenced as described in the RNA approach, by treating the total nucleic acid with DNase prior library preparation. 8 ul of total nucleic acid extracted material from the 6 cervical samples (recommended input for library preparation) were treated with DNase using Turbo DNA-free kit (TermoFisher Scientific, MA, USA) according to manufacturer’s user guide in order to remove DNA.

### Bioinformatics

Indices, included in the Illumina adaptors, were used to assign raw sequence reads obtained from the NextSeq500 (Illumina, San Diego, CA, USA) platform to the originating samples. Demultiplexing was performed using bcl2fastq2 conversion software version 2.19 (Illumina, San Diego, CA, USA). All reads were filtered based on quality and adaptors were trimmed using Trimmomatic version 0.36^24^ with default parameters and 18 bp and 36 bp as minimal read length for RNA and DNA sequenced specimens, respectively. The first 3 nucleotides from every R2 read were trimmed for the RNA sequencing fastq-files using cutadapt version 1.18, as advised within the Smarter stranded total RNA-seq kit used for library preparation.

High-quality paired reads were screened against the human reference genome version GRCh38 using NextGenMap version 0.5.2^25^. The program was run under default settings, except for -i 0.95, -R 0.75 and–silent-clip. Reads that did map to the human reference genome (if they aligned with more than 95% identity over 75% of their length to the human genome) were used to assess sample and protocol adequacy by analyzing presence of reads mapping to the human reference protein coding gene actin beta (ACTB).

High-quality non-human reads were classified using Kraken2 v. 2.1.1^26^, which was run against a reference database containing all RefSeq bacterial and viral genomes (built in December 2020) with a 0.1 confidence threshold. A cut-off of 10 classified reads was used to discriminate positive genera for bacteria and viruses, and results reported all genera which comprised more than 1% of total bacterial or viral reads, respectively.

As HPV is a necessary cause for cervical cancer, we also queried all non-human reads to several HPV databases. Non-human DNA reads were queried against a database of known HPV sequences including all HPV genomes officially established by the International HPV Reference Center (221 officially established HPV types, https://www.hpvcenter.se, accessed on 2020-01-20), together with complete genome sequences from HPV types that are not officially established yet (n = 222, https://pave.niaid.nih.gov, accessed on 2020-01-20), using NextGenMap version 0.5.2^25^ with the same parameters as described previously. Reads that mapped with more than 90% identity over 75% of their length (-i 0.9 -R 0.75) were included for further analysis and subjected to visual inspection using Integrative Genomics Viewer to confirm mapping. Samples were considered positive for HPV (cut-off) if a minimum of 10 reads were detected for any HPV type with at least 90% identity and with an HPV genome coverage of above 10% of that particular HPV type (approximately 750 bp).

Non-human reads from RNA sequencing were queried against all HPV protein sequences included in the PaVE database (Papillomavirus Episteme, accessed on 2019-07-28, including all protein sequences from HPV reference and non-reference genomes), using the open source software Diamond^27^ for protein and translated DNA alignment by blastx with default parameters and –top 1. The same cut-off applied for HPV-positivity (minimum of 10 reads and above 10% genome coverage).

### Ethics approval and consent to participate

Ethical approval was granted by the Regional Ethical Review Board of Stockholm, Sweden (EPN-Dnr: 2014/1242-31/4). Written informed consent was obtained from all participants.

## Results

The metagenome analysis from DNA sequencing of 6 cervical swab samples showed a median number of 138 million (M) high quality reads per sample. The metatranscriptome analysis from RNA sequencing without and with prior DNase-treatment of the extracted genetic material from the swab samples had a median number of 94 M and 46 M high quality reads per sample, respectively (Table 1). However, two of the samples (sample 2 and 5) generated much fewer reads by the RNA sequencing after DNase-treatment (1.4% and 1.7% of the median quality reads) and the quality control by fragment analyses of these two libraries showed no detectable fragments, indicating that the DNase-treatment removed all DNA and that the samples contained very little RNA (Table 1).

**Table 1:** Distribution and classification of high-quality reads among the cervical liquid-based cytology normal and cervical intraepithelial neoplasia 3 (CIN3) swab specimens.

Cyt stage	Sample	Quality reads	Human reads	Human (%)	Bacterial reads	Bacterial (%)	Viral reads	Viral (%)
**DNA**								
CIN3	1	165,909,422	155,445,065	93.69	1,468,818	0.89	16,020	0.010
	2	105,532,360	97,924,315	92.79	648,799	0.61	9957	0.009
	3	139,140,470	128,756,935	92.54	1,281,881	0.92	11,407	0.008
Normal	4	137,252,748	126,670,122	92.29	1,768,777	1.29	14,537	0.011
	5	182,695,608	170,174,824	93.15	997,297	0.55	15,285	0.008
	6	126,899,172	117,964,607	92.96	1,707,273	1.35	12,618	0.010
Median		138,196,609	127,713,529	92.88	1,375,350	0.90	13,577.5	0.010
**RNA**								
CIN3	1	91,008,966	46,873,394	51.50	38,123,009	41.89	3976	0.004
	2	58,353,196	47,974,718	82.21	6,919,203	11.86	3908	0.007
	3	256,490,482	201,377,074	78.51	34,981,758	13.64	17,213	0.007
Normal	4	100,056,316	72,923,611	72.88	17,008,993	17.00	7288	0.007
	5	83,667,632	76,327,134	91.23	1,678,951	2.01	6908	0.008
	6	96,289,626	43,514,776	45.19	46,491,119	48.28	5052	0.005
Median		93,649,296	60,449,165	75.70	25,995,376	15.32	5980	0.007
**RNA with DNase**								
CIN3	1	20,112,832	1,397,079	6.95	17,563,643	87.33	674	0.003
	2	628,262	336,709	53.59	208,256	33.15	42	0.007
	3	113,023,660	11,015,944	9.75	92,860,167	82.16	3676	0.003
Normal	4	144,913,080	7,006,122	4.83	117,826,481	81.31	4034	0.003
	5	776,268	524,911	67.62	176,703	22.76	201	0.026
	6	72,238,864	8,731,751	12.09	59,880,277	82.89	4702	0.007
Median		46,175,848	4,201,601	10.92	38,721,960	81.73	2175	0.005

Cyt stage = Cytology stage, CIN3 = cervical intraepithelial neoplasia stage 3, reads = number of generated raw DNA sequences from the sequencing.

**Figure 1:**
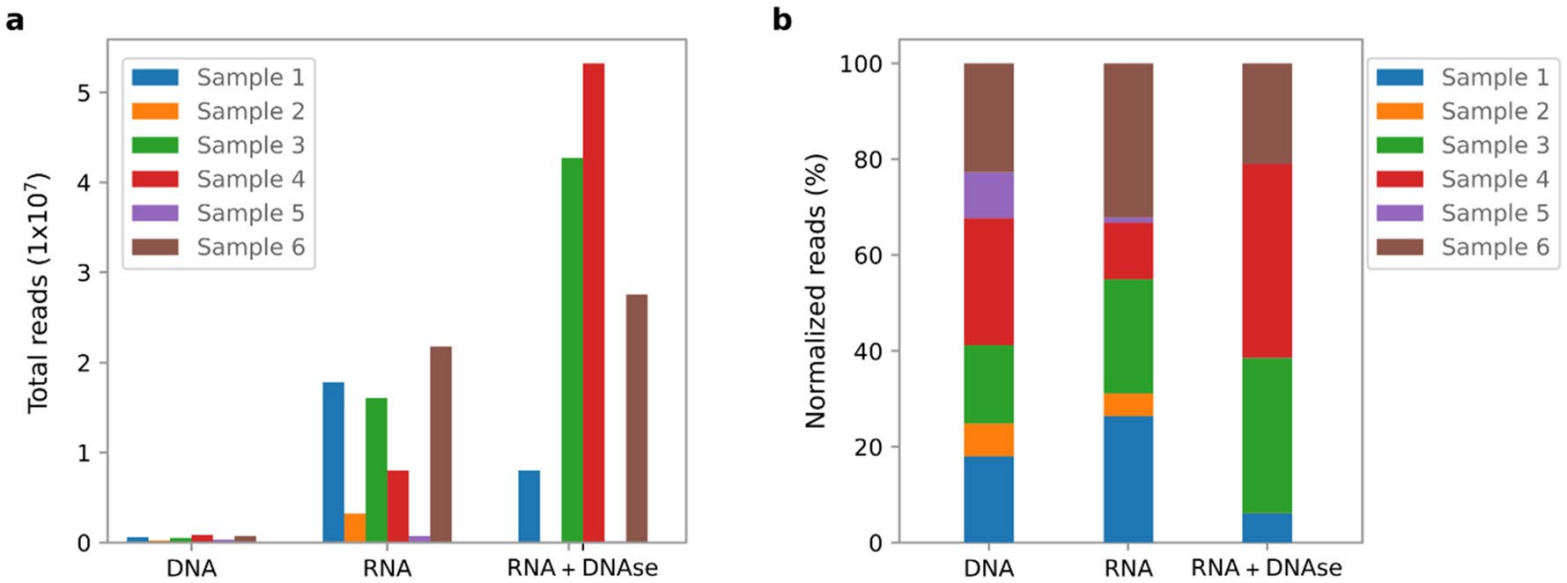
The number of bacterial reads in the 6 cervical swab samples by the 3 sequencing approaches (DNA sequencing, RNA sequencing and RNA sequencing with prior DNase-treatment) (**a**) and the distribution of the reads among the 6 samples normalized for each of the 3 sequencing approaches (**b**). (Image created using Python v. 3.8.5 and Seaborn library v. 0.10.1, https://seaborn.pydata.org/ and Inkscape v. 1.1, https://inkscape.org/).

In agreement with the differences in total read output among the different sequencing approaches, both human and viral reads showed a corresponding decrease in total reads per sample when comparing RNA sequencing output with DNA sequencing output (Table 1). The median relative abundance of annotated human reads was 93% in DNA sequencing, 76% for RNA sequencing and only 11% for RNA sequencing after DNase-treatment (Table 1). The median relative abundance of viral reads followed the human read abundance decrease (0.01%, 0.007% and 0.005% respectively) (Table 1). On the contrary, the annotated bacterial reads increased in relative frequency as well as in total number of reads by RNA sequencing compared to DNA sequencing (Fig. 1a). When comparing the 3 different sequencing approaches, 65% of all annotated bacterial reads were generated by RNA sequencing after DNase-treatment, 33.4% by RNA sequencing without prior DNase-treatment and only 1.6% by DNA sequencing (Fig. 1a). Sample 2 and 5 generated no detectable levels of library fragments by the bioanalyzer after DNase-treatment and hence, very few sequences were obtained, however a more even distribution of bacterial reads among the 6 samples was detected by the DNA sequencing (Fig. 1b). Overall, RNA sequencing with DNase-treatment generated the highest number of annotated bacterial reads per sample (median of 38.7 M reads per sample) as well as the highest relative abundance of reads (median of 82% bacterial reads of the total quality reads), a 91 and 5 times higher relative abundance than the DNA sequencing and RNA sequencing without prior DNase-treatment, respectively (Table 1).

**Figure 2:**
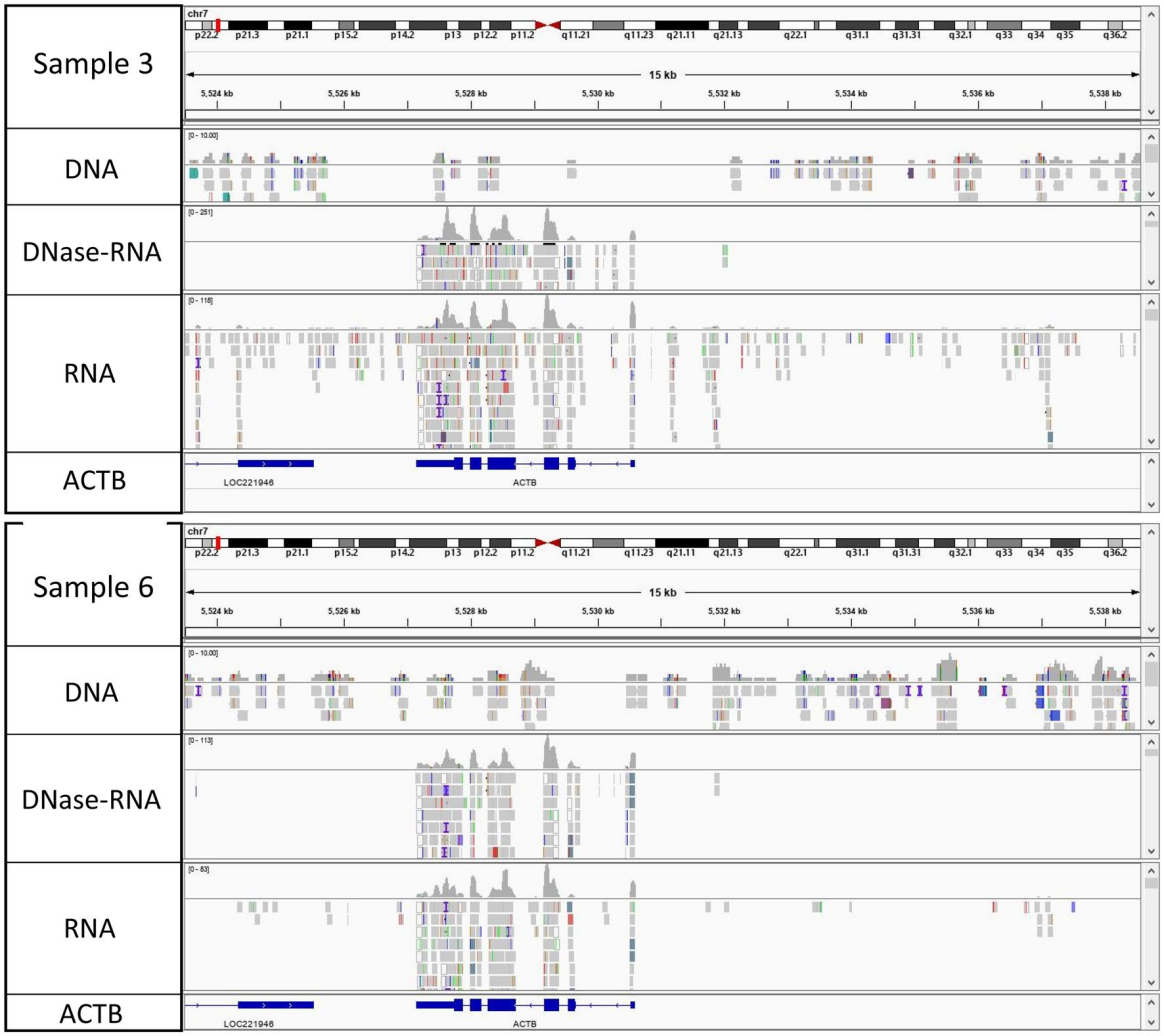
Read distribution and coverage of the human actin beta gene (ACTB) in one CIN3 (sample 3, top panel) and one normal (sample 6, bottom panel) cervical swab by DNA sequencing at the top, RNA sequencing after DNase-treatment in the middle and RNA sequencing without DNase-treatment at the bottom for each of the two samples. The genomic location within chromosome 7 is visualized at the top by a red box and the ACTB gene at the bottom, where the exons are represented by the blue boxes. (Image created using Integrative genomics viewer v. 2.8.13, https://software.broadinstitute.org/software/igv/)^30–32^.

As all 3 sequencing approaches originated from total nucleic acid extracted material (not RNA extracted or DNA extracted material), analysis of the ACTB human gene, which is known to be expressed in cervical cells and commonly used as a reference gene in normalization of RT-qPCR data in cervical cancer cell lines^28^, was performed to investigate presence of DNA in both RNA approaches. Analysis of the ACTB gene showed presence of reads in both introns and exons with the DNA approach, mostly in the exons (with a few reads covering introns) for the RNA approach without prior DNase-treatment, and only reads covering exons for the RNA sequencing with prior DNase-treatment approach, implying that RNA sequencing of the total nucleic acids contains a small fraction of DNA, but almost no DNA left after DNase-treatment prior RNA sequencing (Fig. 2). Even if transcripts cover less than 5% of the human genome^29^, they are much more abundant, highly transcribed genes, as ACTB in cervical cells, may have several 1000’s of copies of a certain transcript in one cell compared to the 2 copies of DNA. Hence, many more reads cover the exon regions compared to introns in RNA sequencing of a total nucleic acids without prior DNase-treatment.

### Cervicovaginal bacteriome

**Figure 3:**
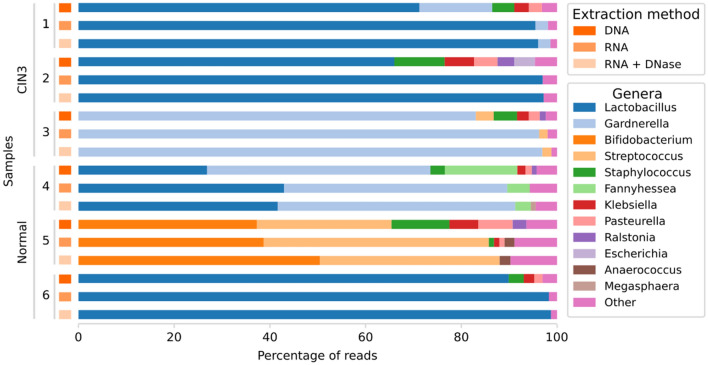
Percentage of reads for bacterial genera for the 6 cervical swab samples by each of the 3 sequencing approaches (DNA sequencing, RNA sequencing and RNA sequencing with prior DNase-treatment). DNA = DNA sequencing, RNA = RNA sequencing, RNA + DNase = RNA sequencing with prior DNase-treatment. CIN3 = cervical intraepithelial neoplasia stage 3, Normal = healthy without neoplasia. Other genera comprise bacterial genera that presented < 1% of the total bacterial reads. (Image created using Python v. 3.8.5 and Seaborn library v. 0.10.1, https://seaborn.pydata.org/ and Inkscape v. 1.1, https://inkscape.org/).

The cervicovaginal bacteriome was analyzed using the Kraken2 program. Bacterial genera with at least 1% of the total number of classified bacterial reads in each of the 18 libraries among the 3 sequencing approaches (DNA, RNA and RNA with prior DNase-treatment) are shown in Fig. 3. Overall, the 3 sequencing approaches corresponded well to each other within each sample, with both RNA sequencing approaches being more similar in the bacteriome patterns of annotated reads compared to DNA sequencing within each sample. In total, 12 bacterial genera were detected with more than 1% of the bacterial reads in at least one sample, with genome sizes ranging from 1.4 to 5.8 Mb (Fig. 3).

Sample 1, 2 and 6 shared a similar bacteriome pattern for the annotated bacterial reads, with *Lactobacillus* genus showing the highest percentage of reads (66% to 99% of the total bacterial reads) for the 3 sequencing approaches. Sample 3 revealed most annotated reads corresponding to *Gardnerella* genus (83% to 97% of the total bacterial reads). In sample 4 most of the annotated reads corresponded to both *Gardnerella* and *Lactobacillus* genera (27% to 50% of the total bacterial reads). Sample 5 showed no reads belonging to the mentioned genera (*Lactobacillus* and *Gardnerella*) and instead, most of the annotated reads belonged to *Bifidobacterium* (37% to 50% of the total bacterial reads) and *Streptococcus* (28% to 47% of the total bacterial reads) (Table 2). *Streptococcus* was also detected in sample 3 (2% to 4% of the total bacterial reads).

**Table 2:** The relative abundance of the annotated bacterial reads for each bacterial genus in percentage of the total number of bacterial reads detected in the 6 cervical swab samples by each sequencing approach (DNA sequencing, RNA sequencing and RNA sequencing with prior DNase-treatment).

Genus	% of total bacterial reads		
	DNA	RNA	DNase
**CIN3—1**			
*Lactobacillus*	71.25	95.47	96.06
*Gardnerella*	15.22	2.62	2.54
*Staphylococcus*	4.59	–	–
*Klebsiella*	3.06	–	–
*Pasteurella*	2.71	–	–
Other*	3.17	1.91	1.40
**CIN3—2**			
*Lactobacillus*	65.99	96.98	97.18
*Staphylococcus*	10.51	–	–
*Klebsiella*	6.16	–	–
*Pasteurella*	4.89	–	–
*Escherichia*	4.33	–	–
*Ralstonia*	3.49	–	–
Other*	4.62	3.02	2.82
**CIN3—3**			
*Gardnerella*	83.04	96.25	96.91
*Staphylococcus*	4.88	–	–
*Streptococcus*	3.74	1.78	1.93
*Klebsiella*	2.45	–	–
*Pasteurella*	2.27	–	–
*Ralstonia*	1.26	–	–
Other*	2.36	1.97	1.17
**Normal—4**			
*Gardnerella*	46.68	46.62	49.62
*Lactobacillus*	26.85	42.96	41.63
*Fannyhessea*	15.14	4.72	3.25
*Staphylococcus*	3.03	–	–
*Klebsiella*	1.72	–	–
*Pasteurella*	1.29	–	–
*Ralstonia*	1.01	–	–
*Megasphaera*	–	–	1.05
Other*	4.28	5.70	4.45
**Normal—5**			
*Bifidobacterium*	37.29	38.71	50.41
*Streptococcus*	28.12	47.04	37.60
*Staphylococcus*	12.09	1.07	–
*Pasteurella*	7.16	1.07	–
*Klebsiella*	6.06	1.13	–
*Ralstonia*	2.86	–	–
*Anaerococcus*	–	2.07	2.25
Other*	6.42	8.91	9.74
**Normal—6**			
*Lactobacillus*	89.91	98.29	98.72
*Staphylococcus*	3.12	–	–
*Klebsiella*	2.18	–	–
*Pasteurella*	1.76	–	–
Other*	3.04	1.71	1.28

DNA = DNA sequencing, RNA = RNA sequencing, DNase = RNA sequencing with prior DNase-treatment. CIN3 = cervical intraepithelial neoplasia stage 3, Normal = healthy without neoplasia. *Other comprises bacterial genera that presented < 1% of total bacterial reads. The genera are sorted from the highest to lowest percentage of the total bacterial reads by DNA sequencing.

*Staphylococcus* was present in all 6 samples from the DNA sequencing at 3% to 12% of the total bacterial reads. On the contrary, RNA sequencing detected *Staphylococcus* in only one sample without the DNase-treatment (1% of total bacterial reads) and in none of the samples with prior DNase-treatment (Table 2). Additional genera with at least 1% of the total bacterial reads from the DNA sequencing were *Klebsiella* and *Pasteurella,* detected in all 6 samples (1% to 7%), *Ralstonia*, detected in 4 of 6 samples (1% to 3%), *Escherichia*, detected in 1 sample (4%) and *Fannyhessea*, detected in 1 sample (15%) (Table 2). Other genera constituted between 2 and 6% of the total bacterial reads, however less than 1% for each individual genus (Table 2). From the two RNA sequencing approaches, it was only *Fannyhessea* of these additional genera that had at least 1% of the total bacterial reads. However, two other genera were detected only by RNA sequencing, *Megasphaera* (1%) and *Anaerococcus* (2%) in one sample each (Table 2).

DNA sequencing detected in total 10 bacterial genera with more than 1% of the total annotated bacterial reads, compared to 9 and 7 genera detected when performing RNA sequencing without and with prior DNase-treatment, respectively (Table 2). In all 6 samples, DNA sequencing detected a higher variety of bacterial genera (between 4 and 7 genera) which contributed to at least 1% of the total bacterial annotated reads, compared to RNA sequencing. By RNA sequencing, the bacterial annotated read distribution was dominated by less than 4 genera for all samples except one sample where the bacterial reads were divided between 6 genera with at least 1% of the total bacterial reads (Table 2).

### Cervicovaginal virome

**Figure 4:**
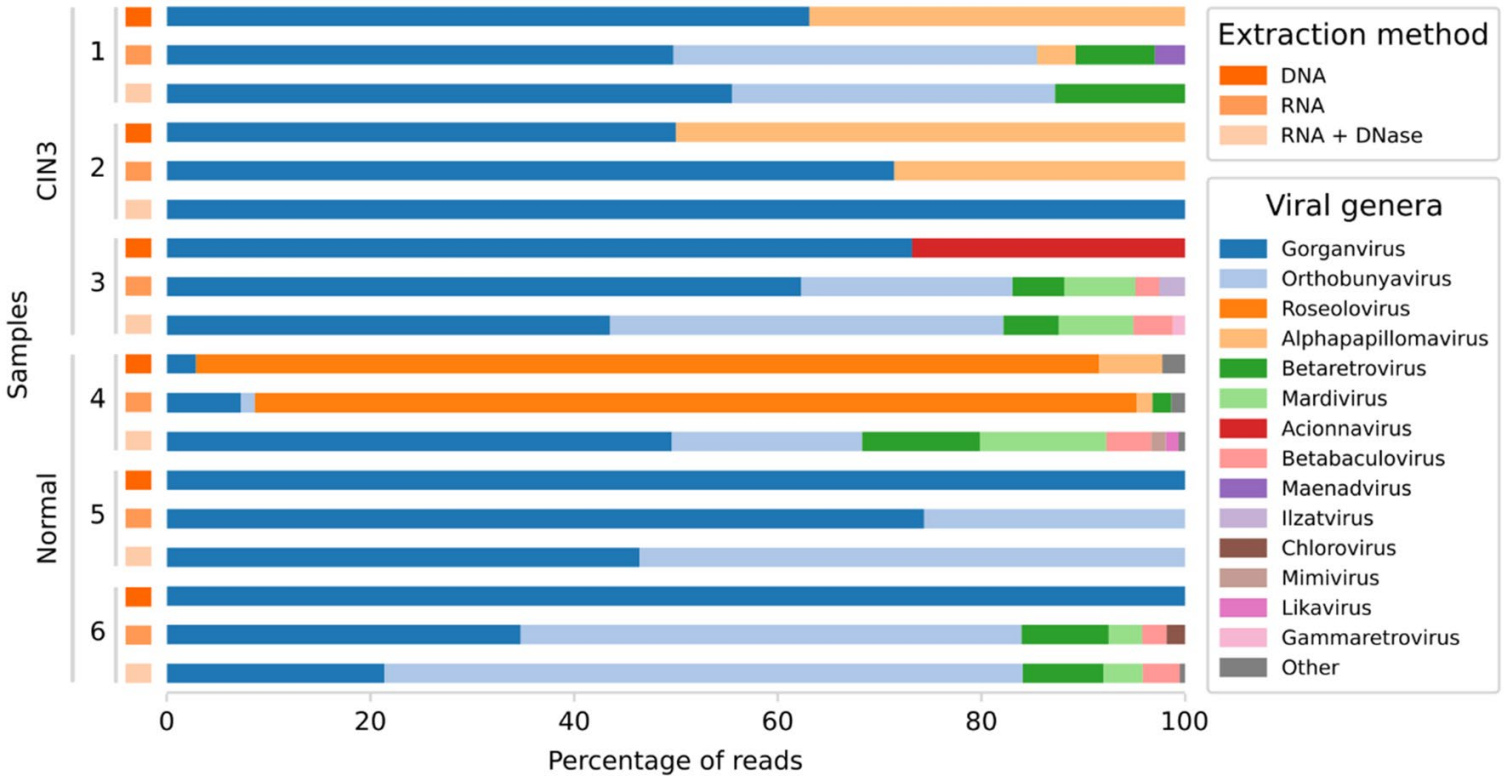
Percentage of reads for viral genera for the 6 cervical swab samples by each of the 3 sequencing approaches (DNA sequencing, RNA sequencing and RNA sequencing with prior DNase-treatment). DNA = DNA sequencing, RNA = RNA sequencing, RNA + DNase = RNA sequencing with prior DNase-treatment. CIN3 = cervical intraepithelial neoplasia stage 3, Normal = healthy without neoplasia. Other genera comprise viral genera that presented < 1% of the total viral reads. (Image created using Python v. 3.8.5 and Seaborn library v. 0.10.1, https://seaborn.pydata.org/ and Inkscape v. 1.1, https://inkscape.org/).

The cervicovaginal virome was analyzed using the Kraken2 program. The viral genera which comprised at least 1% of the total number of annotated viral reads in each of the 18 libraries are shown based on the percentage of reads for the 3 sequencing approaches (DNA, RNA and RNA with prior DNase-treatment) (Fig. 4).

We detected 14 viral genera with at least 1% of the total viral reads whereof 4 genera were detected by DNA sequencing, 10 genera by RNA sequencing and 8 genera by RNA sequencing with prior DNase-treatment (Table 3). One virus genus, Gorganvirus, was present in all 6 samples by all 3 sequencing approaches (Table 3). Gorganvirus belongs to the *Siphoviridae*, a family of viruses with double-stranded DNA genomes of about 50 kb that infects bacteria and archaea, and the family is known to infect the vagina ^4^. In 5 of 6 samples the Gorganvirus genus contributed to most of the viral reads (50% to 100% of the total viral reads) by DNA sequencing. Gorganvirus did also contribute to most of the viral reads in 4 of 6 samples by RNA sequencing without and with DNase-treatment (21% to 100% of the total viral reads) (Table 3). Only one sample (sample 4) had another viral genus that contributed to most of the viral reads, Roseolovirus (89% by DNA sequencing and 87% by RNA sequencing), which was only present in that one sample, but not detected by RNA sequencing with prior DNase-treatment (Table 3). The absence of Roseolovirus reads by the RNA sequencing with prior DNase-treatment implies that the detected reads from the RNA sequencing without prior DNase-treatment correspond to the DNA genome of the Roseolovirus and that the virus is not actively transcribing any of the genes. Roseolovirus genus belongs to *Herpesviridae*, a family of double-stranded DNA viruses with genome size of around 200 kb. Roseolovirus consists of three species that infect humans (*Human betaherpesvirus 6A, 6B and 7)* which are known to infect the mucosal cells of the cervix^33^.

**Table 3:** The relative abundance of the annotated viral reads for each viral genus in percentage of the total number of viral reads detected in the 6 cervical swab samples by each sequencing approach (DNA sequencing, RNA sequencing and RNA sequencing with prior DNase-treatment).

Genus	% of total viral reads		
	DNA	RNA	DNase
**CIN3—1**			
Gorganvirus	63.11	49.73	55.52
Alphapapillomavirus	36.89	3.76	
Orthobunyavirus		35.75	31.72
Betaretrovirus		7.80	12.76
Maenadvirus		2.96	
**CIN3—2**			
Alphapapillomavirus	50.00	28.57	
Gorganvirus	50.00	71.43	100.00
**CIN3—3**			
Gorganvirus	73.21	62.29	43.53
Acionnavirus	26.79		
Orthobunyavirus		20.76	38.62
Mardivirus		6.99	7.33
Betaretrovirus		5.08	5.43
Ilzatvirus		2.54	
Betabaculovirus		2.33	3.88
Gammaretrovirus			1.21
**Normal—4**			
Roseolovirus	88.65	86.59	
Alphapapillomavirus	6.23	1.57	
Gorganvirus	2.88	7.28	49.61
Betaretrovirus		1.84	11.59
Orthobunyavirus		1.36	18.68
Mardivirus			12.37
Betabaculovirus			4.43
Mimivirus			1.43
Likavirus			1.24
Other*	2.23	1.36	0.65
**Normal—5**			
Gorganvirus	100.00	74.36	46.43
Orthobunyavirus		25.64	53.57
**Normal—6**			
Gorganvirus	100.00	34.74	21.39
Orthobunyavirus		49.20	62.68
Betaretrovirus		8.54	7.90
Mardivirus		3.30	3.88
Betabaculovirus		2.39	3.65
Chlorovirus		1.82	
Other*			0.51

DNA = DNA sequencing, RNA = RNA sequencing, DNase = RNA sequencing with prior DNase-treatment. CIN3 = cervical intraepithelial neoplasia stage 3, Normal = healthy without neoplasia. *Other comprises viruses belonging to genera that presented < 1% of the total viral reads and viruses that cannot be classified within a genus. The genera are sorted from the highest to lowest percentage of the total viral reads by DNA sequencing.

Alphapapillomavirus genus consists of small double-stranded DNA viruses with genome size of about 8 kb and includes the causative virus types for CIN3-lesions which are commonly detected in cervical swabs. Alphapapillomavirus was present in 3 of 6 cervical samples (37%, 50% and 6% of the total viral reads) by DNA sequencing and in one additional sample (sample 3), but with less than 1% and therefore not included in the table (Table 3). Alphapapillomavirus was also detected by RNA sequencing in the same 3 samples (4%, 29% and 2% of the total viral reads), but not detected by RNA sequencing with prior DNase-treatment.

The fourth virus genus detected by DNA sequencing was Acionnavirus, another genus of double-stranded DNA bacteriophages within *Myoviridae* known to infect the vagina, detected with 27% of the total viral reads in sample 3, but not detected by RNA sequencing (Table 3). Orthobunyavirus, which is a genus of negative-sense RNA viruses which belong to *Bunyaviridae*—the largest family of RNA viruses with more than 350 isolates—was detected by both RNA sequencing approaches in 5 of 6 samples (not detected in sample 2), where it contributed to most or second most of the viral reads (Table 3). Two additional virus genera with RNA genomes were also detected by RNA sequencing, Betaretrovirus and Gammaretrovirus (Table 3). None of the RNA virus genera were detected by DNA sequencing in any of the samples. RNA sequencing detected 7 additional viral genera with DNA genomes that were not detected by DNA sequencing (Betabaculovirus, Chlorovirus, Ilzavirus, Likavirus, Maenadvirus, Mardivirus and Mimivirus) (Table 3). The genera Ilzavirus, Likavirus, Maenadvirus belong to *Siphoviridae*, a family including Lactobacillus phages, and Mardivirus belongs to *Herpesviridae*. Chlorovirus and Mimivirus are giant DNA viruses that belong to *Phycodnaviridae* and *Mimiviridae*, respectively, and are most probably transients, environmental contaminations or misattributions, as well as Betabaculovirus of the family *Baculoviridae* which infects arthropods.

### HPV detection

HPV types were detected by mapping non-human reads to known HPV nucleotide and protein databases. Both DNA and RNA sequencing approaches detected HPV in all 3 CIN3-samples and in 1 of 3 normal samples, showing a very good concordance between DNA and RNA sequencing (Table 4). The same HPV types were detected by both DNA and RNA sequencing (HPV16, 33, 42, 45, 56, 58, 59 and 67) except for one type (HPV16), detected with a few reads in one of the CIN3-samples only by DNA sequencing (Table 4). When adding DNase-treatment before RNA sequencing there were no HPV reads in any of the 6 samples (Table 4). HPV mapping results are in agreement with Kraken2 taxonomy classification, which found HPV positivity in the same 4 samples. However, one of the positive samples (sample 3) had less than the cutoff at 1% of the total viral reads by Kraken2 classification (only 7 reads of Alphapapillomavirus 7 and 1 read for HPV type 85) and hence, were not included in Table 3.

**Table 4:** Human papillomavirus (HPV) types detected in the 6 cervical swab samples by querying the non-human reads from DNA sequencing, RNA sequencing and RNA sequencing with prior DNase-treatment against HPV databases.

Cyt stage	Sample	HPV types		
		(reads)		
		DNA	RNA	RNA with DNase
CIN3	1	16, 67	67	Neg
		(28, 2247)	(365)	
	2	33	33	Neg
		(2272)	(1588)	
	3	45	45	Neg
		(49)	(108)	
Normal	4	42, 56, 58, 59	42, 56, 58, 59	Neg
		(484, 145, 150, 123)	(226, 254, 133, 758)	
	5	Neg	Neg	Neg
	6	Neg	Neg	Neg

DNA = DNA sequencing, RNA = RNA sequencing, DNase = RNA sequencing with prior DNase-treatment. Cyt stage = Cytology stage, CIN3 = cervical intraepithelial neoplasia stage 3, Normal = healthy without neoplasia, HPV = Human papillomavirus, reads = number of reads mapped for each HPV type (within brackets).

## Discussion

The aim of the study was to compare three different approaches of sequencing of total nucleic acids from cervical swab samples, (i) DNA sequencing, (ii) RNA sequencing and (iii) RNA sequencing with prior DNase-treatment. Analysis of the metagenomes and metatranscriptomes in 18 cervical swab libraries revealed distinct difference in relative detectability between microbial and viral genomes and the human genome. While the number of human reads and its relative abundance decreased when subjecting the samples to RNA sequencing in comparison with DNA sequencing, bacterial genomes generated many more reads in both absolute number of reads as well as in relative abundance. Human DNA depletion is known to favor and optimize the effective detection of bacterial communities by increasing the microbial sequencing depth, especially in clinical samples where approximately 90–95% of metagenomic sequencing reads from samples are annotated as human^10,34–37^. In our study, treatment with DNase I, an endonuclease that cleaves both ssDNA and dsDNA anywhere along the chain, prior to RNA sequencing, decreased the human relative abundance from 76 to 11% and increased the bacterial relative abundance from 15 to 82%, suggesting a higher number of bacterial transcripts compared to human transcripts in the samples, despite more human DNA.

Metagenome analysis from DNA sequencing using Kraken2 program revealed a wider distribution of the bacterial reads to different genera compared to metatranscriptome analysis from RNA sequencing for all samples. In 5 of 6 samples, most of the bacterial reads (about 90%) was generated from only a couple of genera, between 1 and 3 genera had more than 1% of the total bacterial reads, from both RNA sequencing approaches (without and with prior DNase-treatment). By DNA sequencing, between 4 and 7 genera had more than 1% of the total bacterial reads in all 6 samples. One explanation could be that only a few bacteria in each sample contribute to most of the bacterial transcripts, which are sequenced by RNA sequencing. However, several more bacteria are present, but with less transcription activity and hence, detected by DNA sequencing. Despite those differences, the detected bacteriome is overall in concordance between the 3 different sequencing approaches.

Viral metagenome and metatranscriptome analysis revealed the opposite pattern compared to the bacteriome. RNA sequencing generated a wider distribution of viral reads to different genera (10 and 8 genera with more than 1% of the total viral reads by RNA sequencing without and with prior DNase-treatment, respectively) than DNA sequencing (4 genera with more than 1% of the total viral reads). The virus genera detected with most of the viral reads showed a strong concordance between the 3 sequencing approaches, except for the RNA viruses, which were only detected by both RNA sequencing approaches (without and with prior DNase-treatment) and not by DNA sequencing. This fact is also a strong indicator that RNA sequencing of cDNA libraries from total nucleic acids indeed sequence RNA genomes and transcripts.

All the viral genera with DNA genomes that were detected with more than 1% of the total viral reads were double-stranded DNA viruses, but with different genome sizes, from small genomes like Alphapapillomavirus of 8 kb to large genomes like Roseolovirus around 200 kb and giant genomes like Mimivirus of above 1 Mb. Alphapapillomavirus, the known cause of cervical cancer and pre-cancer lesions, was not detected when samples were subjected to DNase-treatment. Therefore, the DNase-treatment should be avoided if the relative abundance or the number of reads of a specific target is expected to be low, as for small virus genomes, and/or if the virus is known to integrate within the human genome, as the DNase will deplete it.

One theoretical advantage of subjecting specimens to RNA sequencing will be the possible detection of RNA viruses, which could be missed when only sequencing the total DNA without prior enrichment for viruses. Indeed, three virus genera with RNA genomes were detected in 5 of the 6 samples by RNA sequencing, but not with DNA sequencing. Furthermore, RNA sequencing provides the result of transcription status, proving that there is an infection. Some viruses (e.g. HPV detected in skin) can be the result of environmental deposition or a transient microbe and DNA detection is in this case not proof of an active infection^10^.

Analysis of detectability of HPV types found few or no differences between DNA and RNA sequencing. While all CIN3 samples showed presence of HPV types, normal samples showed negativity in 2 of 3 samples and one sample being positive for 4 HPV types. The relative read abundance of Alphapapillomavirus of the total viral reads was 37%, 50% and 6% for DNA sequencing and 4%, 29% and 2% for RNA sequencing in the 3 positive samples and less than 1% in one sample by Kraken2 analysis. However, Alphapapillomavirus is a small DNA virus (approximately 8000 bp) with few genes compared to e.g. Roseolovirus (200 kb), which has approximately 25 times larger genome. The 15 times more reads of Roseoloevirus compared to Alphapapillomavirus would actually be the sequencing result of an equal proportion between the two virus genera or even a dominance of the Alphapapillomavirus. *Gardnerella* is known to be a biomarker for HPV progression, whereas abundance of *Lactobacillus* has been associated with clearance of HPV infections^38^. The two samples without HPV infection had no *Gardnerella* detected above 1% of the total bacterial reads. On the contrary, 3 of 4 HPV positive samples had *Gardnerella* reads between 15 and 83% of the total bacterial reads. The detection of *Lactobacillus* showed no difference between normal and CIN3 samples, where 2 of 3 of both normal and CIN3 samples were positive for *Lactobacillus*. However, the study contained only a small pilot of 6 samples, and hence, case–control analysis cannot be conducted.

In conclusion, DNase-treatment of nucleic acids prior RNA sequencing tends to decrease human DNA sequences, but also loose to much of the virome nucleic acids. RNA sequencing generated much more bacterial reads than DNA sequencing and has the advantages of detecting actively transcribed infections, enabling differential gene expression analysis and the possibility to detect RNA genomes. By RNA sequencing, the variety of bacterial genera decreased despite more bacterial reads compared to DNA sequencing, suggesting that just a few of the bacterial genera contribute to most of the bacterial transcripts. More virus genera were detected by RNA sequencing, including both DNA and RNA virus genomes, the latter not possible to detect by DNA sequencing.

## Data Availability

All the aligned non-human sequences are available at the Sequence Read Archive (SRA) with submission number SUB8455397.
